# Distinct Retinal Capillary Plexuses in Normal Eyes as Observed in Optical Coherence Tomography Angiography Axial Profile Analysis

**DOI:** 10.1038/s41598-018-27536-5

**Published:** 2018-06-20

**Authors:** Takao Hirano, Karntida Chanwimol, Julian Weichsel, Tudor Tepelus, Srinivas Sadda

**Affiliations:** 1 Doheny Eye Institute, Los Angeles, California United States; 2 Department of Ophthalmology, Shinshu University School of Medicine, Nagano, Japan; 3 Heidelberg Engineering GmbH, Heidelberg, Germany; 4 Department of Ophthalmology, David Geffen School of Medicine at UCLA, Los Angeles, California United States

## Abstract

Optical coherence tomography angiography (OCTA) allows the retinal microvasculature to be visualized at various retinal depths. Previous studies introduced OCTA axial profile analysis and showed regional variations in the number and location of axially distinct vascular retinal plexuses. OCTA acquisition and processing approaches, however, vary in terms of their resulting transverse and axial resolutions, and especially the latter could potentially influence the profile analysis results. Our study imaged normal eyes using the Spectralis OCT2 with a full-spectrum, probabilistic OCTA algorithm, that, in marked contrast to split-spectrum approaches, preserves the original high OCT axial resolution also within the resulting OCTA signal. *En face* OCTA images are generally created by averaging flow signals over a finite axial depth window. However, we assessed regional OCTA signal profiles at each depth position at full axial resolution. All regions had two sharp vessel density peaks near the inner and outer boundaries of the inner nuclear layer, indicating separate intermediate and deep capillary plexuses. The superficial vascular plexus (SVP) separated into two distinct peaks within the ganglion cell layer in the parafoveal zone. The nasal, superior, and inferior perifovea had a deeper SVP peak that was shifted anteriorly compared to the parafoveal zone. Axial vascular density analysis with high-resolution, full spectrum OCTA thus allows healthy retinal vasculature to be precisely reconstructed and may be useful for clinically assessing retinal pathology.

## Introduction

Optical coherence tomography angiography (OCTA), an imaging technique based on OCT signal variation induced by motion, creates three-dimensional (3-D) reconstructions of retinochoroidal structures without exogenous dye injection^1^. Multiple OCTA studies have qualitatively evaluated common retinochoroidal diseases, including diabetic retinopathy^2^, retinal vein occlusion^3^, and age-related macular degeneration^4^. In addition, quantitative assessments of retinal capillary densities in specific layers in various diseases have been published, utilizing the depth-resolved 3-D OCTA signal^5–7^. The majority of prior OCTA studies have separated the retinal vascular system into two major plexuses, namely the superficial vascular complex (SVC) and the deep vascular complex (DVC). However, early histological studies consistently revealed three distinct retinal capillary plexuses in the human macula, which were named the superficial vascular plexus (SVP), the intermediate capillary plexus (ICP), and the deep capillary plexus (DCP)^8,9^. Furthermore, a radial peripapillary capillary plexus has been shown to run parallel to nerve fiber layer axons that exit the eye via the optic disc^10^.

Visualizing the ICP, SVP, and DCP with OCTA could significantly improve our understanding of retinal diseases, including diabetic retinopathy, macula telangiectasia, and paracentral acute middle maculopathy^11–13^. Recent studies indicated that a novel algorithm for 3-D projection artifact removal (PAR) may provide improved visualization of all three parafoveal retinal capillary plexuses, including the ICP^14^. Additionally, Campbell *et al*.^15^ examined local axial profiles of projection-removed split-spectrum amplitude decorrelation (SSADA) OCTA signals at various retinal positions. Splitting the spectrum is an effective approach for reducing noise and increasing the quality of the flow signal, but involves a loss in axial resolution. This reduction in axial resolution could potentially impact the assessment of the separation of distinct retinal vascular layers, particularly in scenarios where the layers are closely spaced in the axial direction. In addition, 3-D PAR could also impact the observed axial flow profiles. They detected peaks in capillary density that corresponded to the capillary plexuses in the peripapillary, parafoveal, perifoveal, and peripheral regions. While four distinct vascular peaks were distinguished in the peripapillary region and these peaks were associated with two distinct vascular layers within each of the SVC and DVC, these two distinct superficial vascular layers apparently merged in the parafoveal and perifoveal zones. In addition, the two distinct layers within the DVC could not be separated or distinguished in more peripheral regions of the retina.

To further assess the potential impact of axial resolution and 3-D PAR, we performed axial profile analysis on the high-resolution signal from full-spectrum, probabilistic OCTA data, with and without 3-D PAR, and evaluated the segregation of the vascular layers. This full-spectrum OCTA processing preserves the original high OCT axial resolution within the OCTA signal and thus provides an ideal basis for subsequent axial profile analysis.

## Results

### Human Subjects

This prospective, cross-sectional study included 22 normal subjects (14 male, 8 female) with a mean age ± standard deviation of 34.0 ± 6.9 years (range: 22–55 years).

### Impact of 3-D PAR on axial vascular density profile analysis

**Figure 1:**
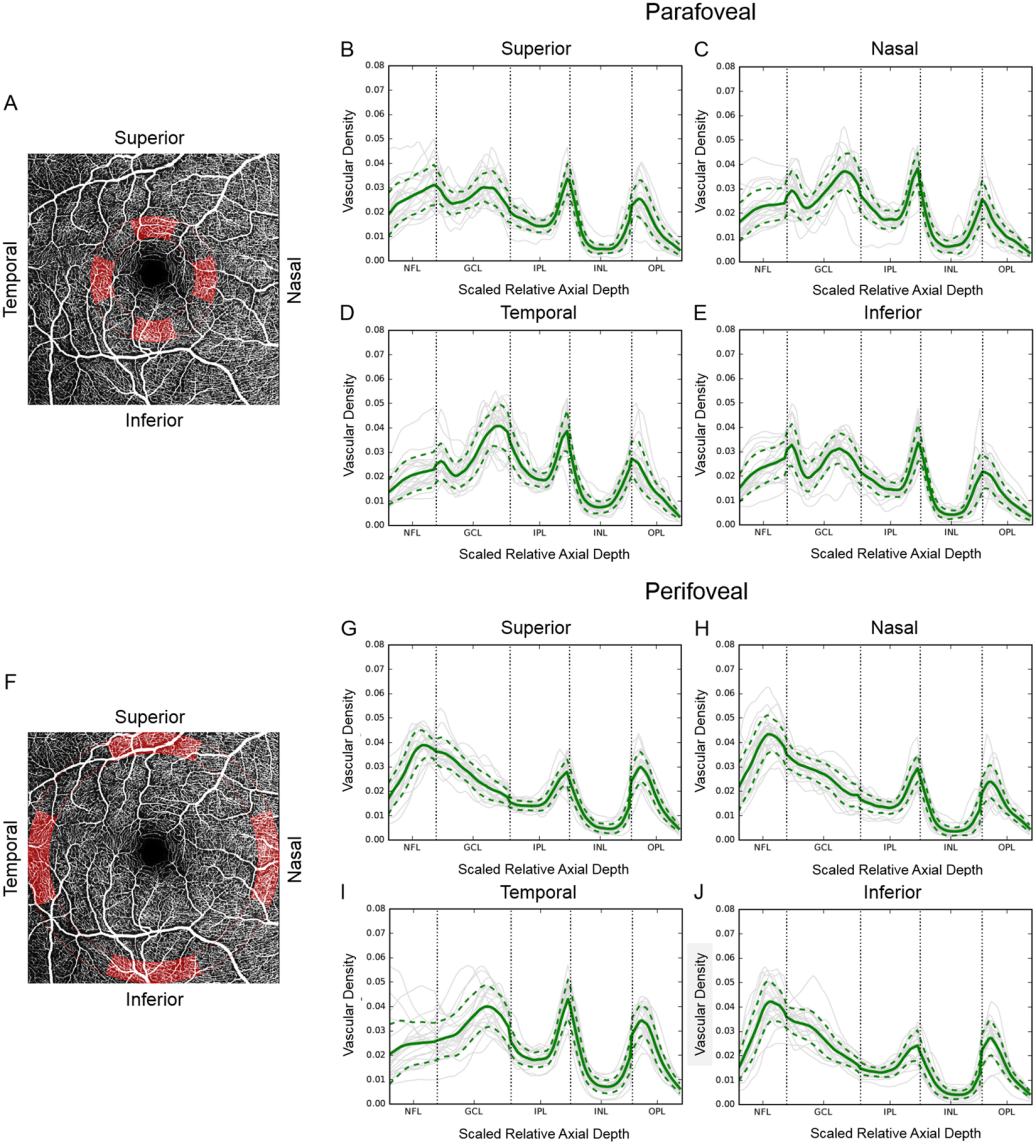
Parafoveal and perifoveal axial vascular density profiles (vascular density vs. scaled relative axial depth) obtained with optical coherence tomography angiography. An *en face* image is also shown for reference (**A**,**F**). Profiles in parafoveal region (2.5°–3.75° radial distance to the foveal center) were obtained from the superior (**B**), nasal (**C**), temporal (**D**), and inferior (**E**) quadrants (red shaded regions in **A**). Profiles in the perifoveal region (6.25°–7.5° radial distance to the foveal center) were obtained from the superior (**G**), nasal (**H**), temporal (**I**), and inferior (**J**) quadrants (red shaded regions in **F**). Individual patient profiles are shown as grey solid lines and mean density is shown as a green solid line. The green dotted lines represent one standard deviation above and below the mean. Sharp peaks, corresponding to the intermediate and deep capillary plexuses, are apparent near the inner and outer INL borders in all examined parafoveal regions. The superficial vascular plexus contained both a small peak at the NFL-GCL junction and a larger, broader peak within the GCL. In all four perifoveal regions, sharp peaks corresponding to the intermediate and deep capillary plexuses are apparent near the inner and outer INL borders in all examined regions. Unlike in the parafoveal region, the SVP was not comprised of two distinct peaks. The tall SVP peak was shifted towards the ILM in the nasal, superior, and inferior quadrants. NFL: nerve fiber layer; GCL: ganglion cell layer; IPL: inner plexiform layer; INL: inner nuclear layer; OPL: outer plexiform layer.

**Figure 2:**
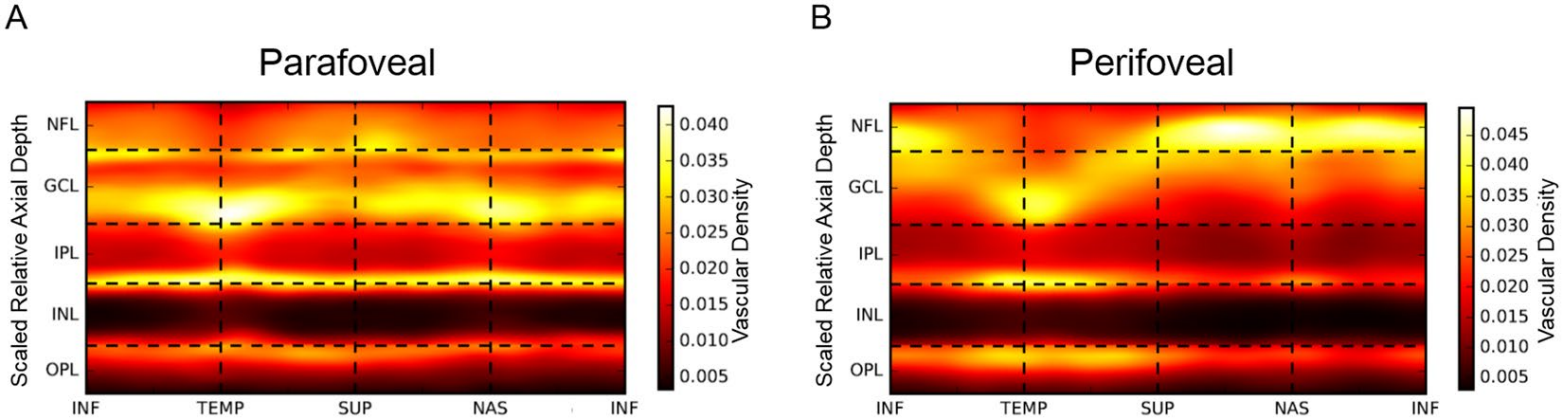
Continuous annular vascular density heat maps of the parafoveal and perifoveal rings. The average axial OCTA signal profile over all 22 eyes is plotted against the orientation within the para- or perifoveal rings. Four vessel layers, represented as white, “hot” bands, are apparent at a relatively consistent axial location in the parafovea (**A**). In contrast, only three vessel layers are distinguishable in the perifovea (**B**). INF: inferior, TEMP: temporal, SUP: superior, NAS: nasal.

**Figure 3:**
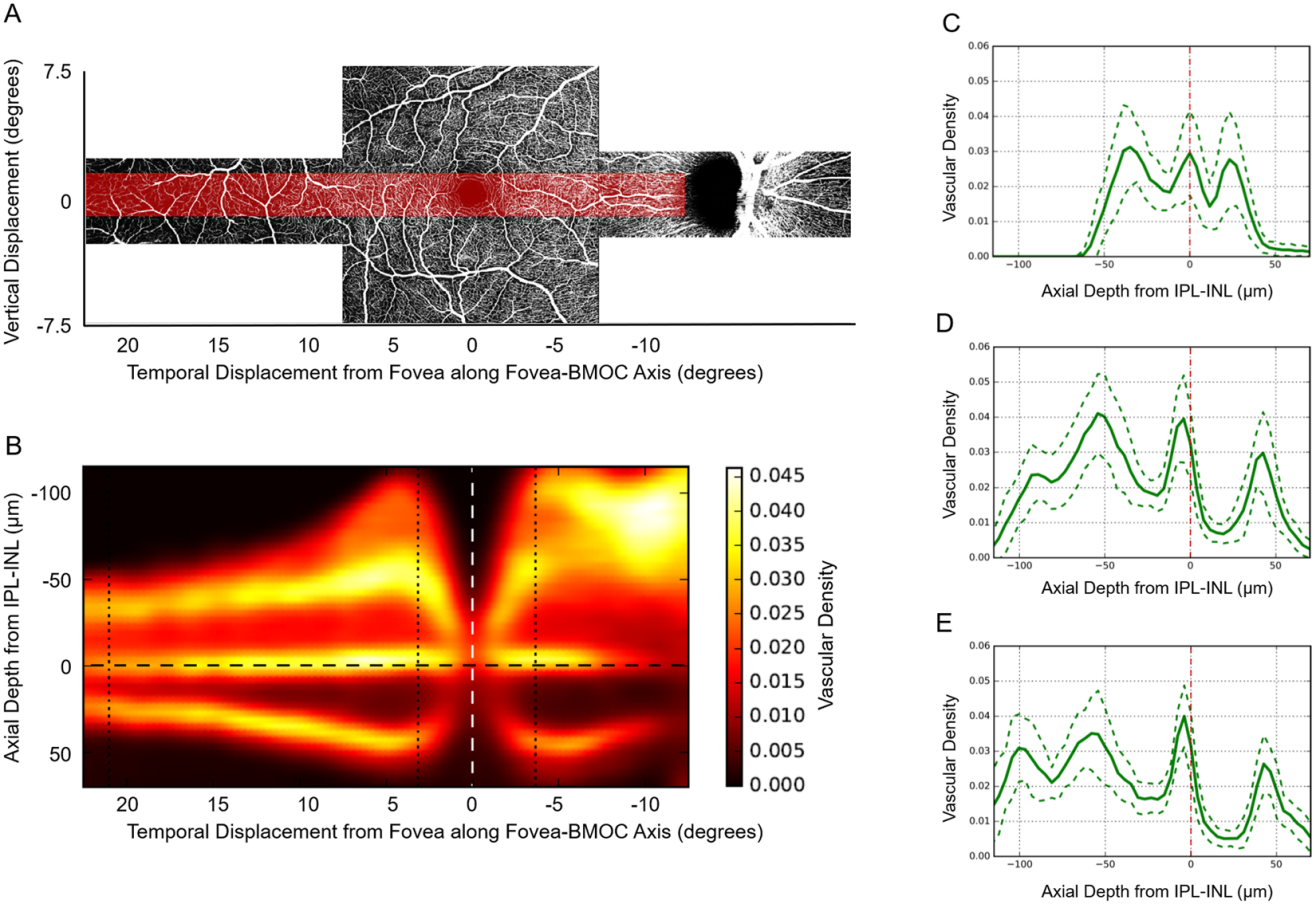
Vascular density heat map plotted along the fovea-BMOC axis. The top *en face* image (**A**) is a montage of one foveal-centered 15° × 15° OCTA scan and two 15° × 5° OCTA scans displaced temporally and nasally by 15° along the Fovea–BMOC axis. The red shaded area in (**A**) corresponds to where the vascular density map (**B**) was obtained. The vascular density measurement band was 2.5° wide and centered on the fovea-BMOC axis. Vascular density was measured between 22.5° temporal and 12.5° nasal. Axial vascular density profiles (vascular density vs. axial depth from IPL-INL) were obtained at the left (**C**), middle (**D**) and right (**E**), black dotted line in (**B**). The SVP, ICP, and DCP are visible throughout the vascular density heat map, including in far temporal locations. A fourth plexus in the nerve fiber layer is also detectable at approximately 5°, both nasally and temporally from the fovea. BMOC: Bruch’s membrane opening center, OCTA; optical coherence tomography angiography; SVP: superficial vascular plexus; ICP: inner capillary plexus; DCP: deep capillary plexus; SVP: superficial vascular plexus; IPL: inner plexiform layer, INL: inner nuclear layer.

Results of the OCTA axial profile analysis with 3-D PAR are presented in the main text of this article (Figs 1, 2 and 3), while the corresponding results without 3-D PAR are qualitatively similar and are therefore included as supporting information to this article for comparison (Figs S1, S2 and S3, respectively).

### Axial vascular density profiles

Axial vascular density profiles were clearly defined in both the parafoveal (2.50°–3.75° from foveal center, Fig. 1A–E) and perifoveal (6.25°–7.50° from foveal center, Fig. 1F–J) zones. All eight examined regions around the fovea had sharp vessel density peaks near the inner and outer boundary of the inner nuclear layer (INL), which represented the ICP and DCP. However, the SVP location varied by region. In all four parafoveal regions, the SVP appeared as two peaks, with one small peak at the nerve fiber layer (NFL)-ganglion cell layer (GCL) junction and one larger, broader peak within the GCL. In the perifoveal region, a single SVP peak was observed. Additionally, nasal, superior, and inferior perifoveal regions had a tall SVP peak that was shifted towards the inner limiting membrane (ILM).

### Annular vascular density heat maps

Regional variations in retinal vascular density could also be clearly appreciated in continuous parafoveal and perifoveal annular vascular density heat maps, where individual microvasculature layers are evident as “hot” bands (Fig. 2). Four vessel layers with a relatively consistent axial location were observed in the parafoveal retina. In contrast, only three vessel layers were observed in the perifoveal retina, with a displacement away from the ILM observed in the temporal region.

### Fovea-BMOC vascular density heat map

The axial vascular density heat map along the fovea-Bruch’s membrane opening center (BMOC) axis demonstrates the clear separation of SVP, ICP, and DCP from a region 12.5° nasal to 22.5° temporal to the fovea (Fig. 3A,B). It should be noted that the ICP and DCP did move closer together in the temporal retina, but remained distinct vascular layers on high axial resolution OCTA images (Fig. 3C). The fourth plexus in the NFL, which was already visible in the parafoveal regions in Figs 1 and 2, could also be detected at approximately 3–5° from the fovea, both temporally and nasally (Fig. 3D,E, respectively). Therefore, our observation of the additional resolvable parafoveal fine structure within the superficial vascular complex does not strongly depend on the specific routine that is applied to register individual A-Scans here, compared with the previous Figs 1 and 2.

### *En face* OCTA images of vascular plexuses

**Figure 4:**
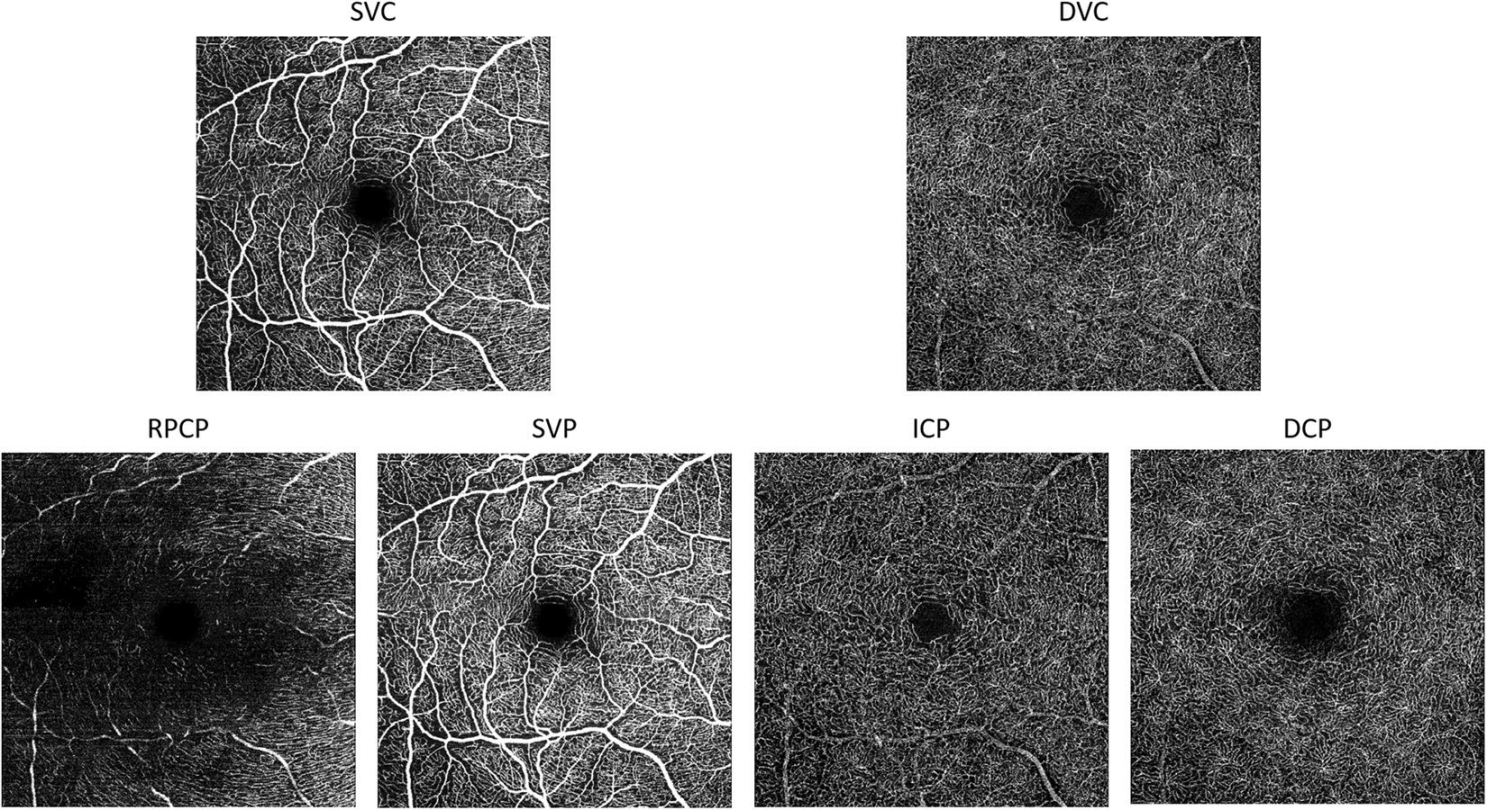
*En face* macular OCTA images of vascular plexuses identified with axial profile analysis in a healthy right eye. En face OCTA vascular layers are exported from the Spectralis software after application of projection artefact removal in the deeper layers, DVC, ICP, and DCP. The SVP had centripetal branches that terminated in a capillary ring around the foveal avascular zone. *En face* OCTA images of the DVC could be divided into *en face* images of the ICP and DCP, which had distinct vascular morphologies. A vortex-like configuration of vascular loops surrounded a central seed point (single point surrounded by yellow dotted lines) was apparent in the DCP, but not in the ICP. OCTA: optical coherence tomography angiography; PAR: projection artifact removal, SVC: superficial vascular complexes, DVC: deep vascular complexes, SVP: superficial vascular plexus, ICP: intermediate capillary plexuses, DCP: deep capillary plexuses.

*En face* OCTA images were generated to evaluate differences in vascular plexus morphology. Images were created based on the vascular layers identified by OCTA axial profile analyses (Fig. 4). *En face* OCTA images of the SVP revealed a centripetal branching pattern that terminated in a capillary ring around the foveal avascular zone. Additionally, *en face* OCTA images of the DVC could be divided into the ICP and DCP, each of which had a distinct vascular morphology. Most notably, a vortex-like configuration of vascular loops surrounding a central seed point was apparent in the DCP, but not in the ICP.

## Discussion

Studies using SSADA OCTA with 3-D PAR and axial profile analysis have provided insight into the 3-D architecture of the retinal microcirculation^14,15^. Although splitting the spectrum improves the signal-to-noise ratio of flow detection, it induces some axial resolution loss compared to full-spectrum OCTA algorithms^16^. The current study demonstrated that axial vascular density analyses obtained with high resolution signals from full-spectrum, probabilistic OCTA provides consistent visualization of all three retinal capillary plexuses, including the ICP, in the macula of healthy eyes. All examined macular regions had two sharp vessel density peaks near the inner and outer INL boundaries, corresponding to the ICP and DCP, respectively. This finding is in agreement with prior immunohistochemical^8^ and OCTA^15^ studies. Unlike the ICP and DCP, the location of the SVP significantly varied among retinal regions. In all parafoveal regions examined (including the temporal region), the SVP had both a small peak at the NFL-GCL junction and a larger, broader peak within the GCL. This separation of the parafoveal SVP into two layers was not observed in prior SSADA OCTA-based analyses. We speculate that this discrepancy may have resulted from differences between full-spectrum and split-spectrum approaches. In contrast to the parafovea, only a single SVP peak was observed in the perifoveal region. This peak was shifted towards the ILM in the nasal, superior, and inferior quadrants, presumably because of the thicker NFL associated with the arcuate and papillo-macular bundles in these quadrants^17^.

Distinct ICP and DCP vascular morphologies were observed using our high-axial resolution approach. Only the DCP had vascular loops and tufts, which appeared to be radially-organized around a central point which appeared to be a vertical inter-connecting vessel. These findings differ from those of previous studies, in which the ICP and DCP appeared quite similar. We again wonder if this discrepancy resulted from differences in axial resolution, though it may also reflect differences in the underlying OCTA data/signal or projection removal techniques. Regardless, the difference in morphology and the sharp and distinct peaks of the ICP and DCP would argue that they should not be combined as a deep vascular complex. The very clear and consistent separation between the ICP and DCP was readily apparent on continuous heat maps of the parafoveal and perifoveal rings, as well as along the fovea –BMOC axis. Although the ICP and DCP moved closer together at further distances temporal to the fovea, unlike in previous reports^15^, even at 22.5° temporal to the fovea, the ICP and DCP could be clearly separated at a peak to peak distance of roughly 25 µm. These findings indicate that shifting the outer boundary of the inner plexiform layer (IPL) by constant amounts, either anteriorly or posteriorly, yields practical approximations for the separating boundaries between SVP and ICP, as well as ICP and DCP, respectively.

Of note, we observed similar results with and without 3-D PAR, with regards to the number and axial position of the peaks, though the prominence of the peaks was clearly enhanced by PAR. Nonetheless, the similarity of the findings with and without 3-D PAR highlights the robustness of our results.

Our study, however, is not without limitations. Our sample size was relatively small and largely included younger subjects with an average age of 34 years. A much larger study population with a wider age range is needed to establish a normative database of axial vascular density. Such a study is needed to fully understand the impact of age, gender, race, refractive error, and axial length on vascular density profiles. Additionally, our study only examined healthy subjects. Studies that include subjects with retinal and systemic disorders are also needed to understand how common conditions affect axial vascular density profiles. Finally, although it would have been ideal to compare SSADA and the full-spectrum probabilistic method applied to the same raw OCTA data, the OCTA processing scheme chosen by each manufacturer may be optimized to their strategy for acquisition and the performance of their machine. Thus, such a comparison is potentially confounded and may be difficult to interpret.

In summary, axial vascular density analysis of the high-resolution signal from full-spectrum OCTA allows for the precise reconstruction of the 3-D organization of the retinal microvasculature in healthy eyes.

## Methods

This cross-sectional, observational study was reviewed and approved by the Institutional Review Board of the University of California, Los Angeles (UCLA). All study conduct adhered to the tenets of the Declaration of Helsinki and written informed consent was obtained from all participants.

### Study Population

All subjects were recruited at the Doheny-UCLA Eye Centers, and 22 right eyes of 22 subjects were enrolled. Subjects were only included if they were healthy and had normal eyes. Subjects were excluded if any of the following were present: significant refractive error (myopia more severe than −6.00 diopters or hyperopia more severe than +3.00 diopters), ocular media opacity, or retinal disease in the study eye as assessed by dilated ophthalmoscopy.

### Image Acquisition and Processing

The right eye of each subject underwent OCTA imaging using a Spectralis OCT2 (Heidelberg Engineering, Heidelberg, Germany) with 880 nm central wavelength, 7 µm OCT resolution in tissue, 85 kHz A-Scan rate, and 1.2 mW incident optical power. The native OCTA acquisition software of the device (version 6.9.2.700) utilizes a full-spectrum, probabilistic OCTA algorithm. This algorithm avoids spectral splitting during signal reconstruction to preserve the original high optical axial resolution of the underlying OCT signal in the resulting OCTA data. It associates a probability of flow, with respect to an underlying statistical model for OCT signal fluctuations over time, to each voxel.

Macular OCTA scans were captured with an isotropic lateral scan density of 768 A-scans per 15° (approximately 6 µm per A-Scan). Three scans were obtained, one foveal-centered 15° × 15° scan and two 15° × 5° scans displaced temporally and nasally by 15° along the fovea–BMOC axis. The OCTA signal at each depth position was assessed locally at full-axial resolution (i) in 8 regions around the fovea consisting of superior, temporal, inferior, and nasal areas in both parafoveal (2.5°–3.75°) and perifoveal (6.25°–7.5°) zones, as well as, (ii) continuously along the fovea–BMOC axis.

To focus the analysis on the evaluation of the microvasculature (i.e. capillary circulation), the largest superficial vessels were automatically identified, segmented and excluded from the axial OCTA profile analysis. Here we employed a commonly used strategy based on the eigenvalue analysis of multiscale Hessian image filter responses^18^ and subsequent thresholding. Briefly, from each OCTA volume cube we generated a 2-D *en* face projection by axial integration of the OCTA signal between the ILM and the IPL-INL interface. Large vessels on this enface projection were highlighted using a 2-D multiscale vesselness filter^18^, with a minimum cutoff length scale of 5 pixels (approximately 28.5 µm). The brightest 9% pixels were assumed to represent large superficial vessels. Consequently, the complete axial signal (i.e. whole A-Scans) at those identified locations, consisting of large superficial vessels and their prominent subjacent shadow regions in OCTA, was excluded from the subsequent axial signal analysis.

After automated removal of the largest superficial vessels by the above method, the flow signal in individual A-Scans was axially aligned by either rescaling individual retinal layers to their uniform average thickness (for results in Figs 1, 2), or by a rigid axial shift to a uniform reference location defined by the posterior IPL boundary (for results in Fig. 3). Although the latter rigid transformation approach does not compensate for inter-eye variability in retinal layer thickness and thus causes some blurring of the vascular peaks at axial positions further away from the reference location, it preserves the original scaling of axial depth in physical units (i.e. µm) and thus allows quantification of the spatial separation of axially adjacent vascular layers in these absolute units. Subsequently aligned A-Scans were averaged within a narrow lateral window to generate a vascular profile at each axial position in the vascular retina (from the ILM through the outer plexiform layer). Specifically, averaged regions that are highlighted in Fig. 1A and F, for computing vascular axial profiles in Fig. 1B–E and G–J spread over 64 A-Scans radially and approximately 126 A-Scans or 276 A-Scans angularly, respectively. The corresponding heat maps in Fig. 2A,B were averaged over 64 A-Scans radially and smoothed angularly using a Gaussian low-pass filter with standard deviation of approximately 28 A-Scans and 61 A-Scans, respectively. The resulting heat map in Fig. 3B was averaged over 128 A-Scans perpendicularly to the Fovea to BMOC axis, then low-pass filtered tangentially to this axis with a Gaussian filter (standard deviation, 30 A-Scans). In general, our results were robust against changes in the specific size of the averaging window, given that it was sufficiently larger than the typical microvascular spacing and simultaneously sufficiently small to preserve the observed regional variations among different retinal locations.

These axial profiles were generated before and after the application of 3-D PAR which was performed using the method described in previous reports^14,15,19^. As this particular approach to PAR was developed based on an amplitude decorrelation OCTA signal and because we did not find a significant correlation of the OCTA signal from probabilistic full-spectrum OCTA with OCT signal amplitude, the normalization of the OCTA signal with log amplitude was omitted. Aside from this modification, our approach was identical to that of Jia *et al*.^16^. Results of the OCTA axial profile analysis with 3-D PAR are presented within the main text of this article, while the corresponding results without 3-D PAR are included as supporting information for comparison (Figs S1, S2 and S3).

## Electronic supplementary material

Supplemental figures

## References

[1] Makita S, Hong Y, Yamanari M, Yatagai T, Yasuno Y (2006). Optical coherence angiography. Opt Express.

[2] Ishibazawa A (2016). Characteristics of Retinal Neovascularization in Proliferative Diabetic Retinopathy Imaged by Optical Coherence Tomography Angiography. Invest Ophthalmol Vis Sci.

[3] Kashani AH, Lee SY, Moshfeghi A, Durbin MK, Puliafito CA (2015). Optical Coherence Tomography Angiography of Retinal Venous Occlusion. Retina.

[4] Moult E (2014). Ultrahigh-speed swept-source OCT angiography in exudative AMD. Ophthalmic Surg Lasers Imaging Retina.

[5] Al-Sheikh M, Akil H, Pfau M, Sadda SR (2016). Swept-Source OCT Angiography Imaging of the Foveal Avascular Zone and Macular Capillary Network Density in Diabetic Retinopathy. Invest Ophthalmol Vis Sci.

[6] Ghasemi Falavarjani K (2017). Optical Coherence Tomography Angiography Analysis of the Foveal Avascular Zone and Macular Vessel Density After Anti-VEGF Therapy in Eyes With Diabetic Macular Edema and Retinal Vein Occlusion. Invest Ophthalmol Vis Sci.

[7] Kim AY (2016). Quantifying Microvascular Density and Morphology in Diabetic Retinopathy Using Spectral-Domain Optical Coherence Tomography Angiography. Invest Ophthalmol Vis Sci.

[8] Tan PE (2012). Quantitative confocal imaging of the retinal microvasculature in the human retina. Invest Ophthalmol Vis Sci.

[9] Tan PE, Yu PK, Cringle SJ, Yu DY (2014). Quantitative assessment of the human retinal microvasculature with or without vascular comorbidity. Invest Ophthalmol Vis Sci.

[10] Henkind P (1967). Radial peripapillary capillaries of the retina. I. Anatomy: human and comparative. Br J Ophthalmol.

[11] Park JJ, Soetikno BT, Fawzi AA (2016). Characterization of the Middle Capillary Plexus Using Optical Coherence Tomography Angiography in Healthy and Diabetic Eyes. Retina.

[12] Thorell MR (2014). Swept-source OCT angiography of macular telangiectasia type 2. Ophthalmic Surg Lasers Imaging Retina.

[13] Rahimy E, Kuehlewein L, Sadda SR, Sarraf D (2015). Paracentral Acute Middle Maculopathy: What We Knew Then and What We Know Now. Retina.

[14] Garrity ST, Iafe NA, Phasukkijwatana N, Chen X, Sarraf D (2017). Quantitative Analysis of Three Distinct Retinal Capillary Plexuses in Healthy Eyes Using Optical Coherence Tomography Angiography. Invest Ophthalmol Vis Sci.

[15] Campbell JP (2017). Detailed Vascular Anatomy of the Human Retina by Projection-Resolved Optical Coherence Tomography Angiography. Sci Rep.

[16] Jia Y (2012). Split-spectrum amplitude-decorrelation angiography with optical coherence tomography. Opt Express.

[17] Li ST, Wang XN, Du XH, Wu Q (2017). Comparison of spectral-domain optical coherence tomography for intra-retinal layers thickness measurements between healthy and diabetic eyes among Chinese adults. PLoS One.

[18] Frangi AF, Niessen WJ, Vincken KL, Viergever MA (1998). Multiscale vessel enhancement filtering. Lecture Notes in Computer Science.

[19] Zhang M (2016). Projection-resolved optical coherence tomographic angiography. Biomed Opt Express.

