# Supplementary material for: Distinct Retinal Capillary Plexuses in Normal Eyes as Observed in Optical Coherence Tomography Angiography Axial Profile Analysis
Source: Sci Rep. 2018 Jun 20;8:9380. doi: 10.1038/s41598-018-27536-5 (PMC6010462; doi:10.1038/s41598-018-27536-5)

Supplemental Data for:

Distinct Retinal Capillary Plexuses in Normal Eyes as Observed in Optical Coherence Tomography Angiography Axial Profile Analysis

Takao Hirano[1,2,4], Karntida Chanwimol[1,4], Julian Weichsel[3], Tudor Tepelus[1], Srinivas Sadda[1,4]

[1]Doheny Eye Institute, Los Angeles, California, United States
[2]Department of Ophthalmology, Shinshu University School of Medicine, Nagano, Japan
[3]Heidelberg Engineering GmbH, Heidelberg, Germany
[4]Department of Ophthalmology, David Geffen School of Medicine at UCLA, Los Angeles, California, United States

Supplemental Figure Legends

Supplemental Figures 1-3

Supplemental Figure Legends

Supplemental Figure 1. Parafoveal and perifoveal axial vascular density profiles obtained with optical coherence tomography angiography without 3-D PAR (vascular density vs. scaled relative axial depth). An en face image is also shown for reference (A, F). Profiles in parafoveal region (2.5°–3.75° radial distance to the foveal center) were obtained from the superior (B), nasal (C), temporal (D), and inferior (E) quadrants (red shaded regions in A). Profiles in perifoveal region (6.25°–7.5° radial distance to the foveal center) were obtained from the superior (G), nasal (H), temporal (I), and inferior (J) quadrants (red shaded regions in F). Individual patient profiles are shown as grey solid lines and mean density is shown as a green solid line. The green dotted lines represent one standard deviation above and below the mean. Sharp peaks, corresponding to the intermediate and deep capillary plexuses, are apparent near the inner and outer INL borders in all examined parafoveal regions. The superficial vascular plexus contained both a small peak at the NFL-GCL junction and a larger, broader peak within the GCL. In all 4 perifoveal regions, sharp peaks corresponding to the intermediate and deep capillary plexuses are apparent near the inner and outer INL borders in all examined regions. Unlike in the parafovea, the SVP was not made up of two distinct peaks. The tall SVP peak was shifted towards the ILM in the nasal, superior, and inferior quadrants. NFL: nerve fiber layer, GCL: ganglion cell layer, IPL: inner plexiform layer, INL: inner nuclear layer, OPL: outer plexiform layer, PAR: projection artifact removal

Supplemental Figure 2. Continuous annular vascular density heat maps of the parafoveal and perifoveal rings. The average axial OCTA signal profile without 3-D PAR over all 22 eyes is plotted against the orientation within the para- or perifoveal rings. Three vessel layers were distinguishable in the parafovea (A) and the perifovea (B). INF: inferior, TEMP: temporal, SUP: superior, NAS: nasal, PAR: projection artifact removal

Supplemental Figure 3. Vascular density heat map plotted along the fovea-BMOC axis. The top *en face* image (A) was a montage of one foveal-centered 15°x15° OCTA scan and two 15°x5° OCTA scans without 3-D PAR displaced temporally and nasally by 15° along the fovea-BMOC axis. The red shaded area in (A) corresponds to where the vascular density map (B) was obtained. The

vascular density measurement band was 2.5° wide and centered on the fovea-BMOC axis. Vascular density was measured between 22.5° temporal and 12.5° nasal. Axial vascular density profiles (vascular density vs. axial depth from IPL-INL) were obtained at the left (C), middle (D) and right (E) black dotted line in (B). The SVP, ICP, and DCP were visible throughout the vascular density heat map, including in far temporal locations. A fourth plexus in the nerve fiber layer was not detected. BMOC: Bruch's membrane opening center, OCTA: optical coherence tomography angiography, SVP: superficial vascular plexus, ICP: inner capillary plexus, DCP: deep capillary plexus, SVP: superficial vascular plexus, IPL: inner plexiform layer, INL: inner nuclear layer, PAR: projection artifact removal

Supplemental Figure 1

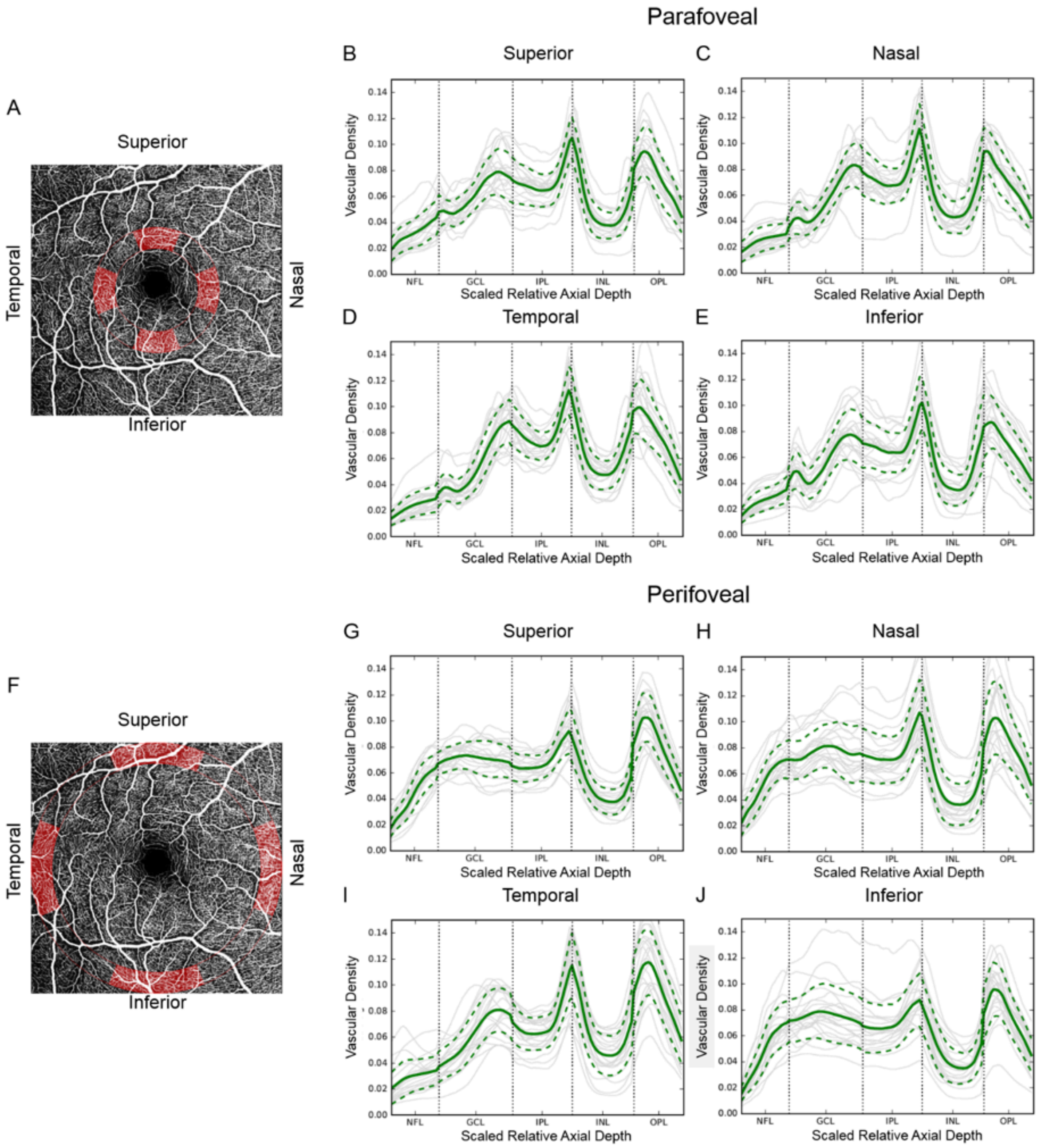

Supplemental Figure 2

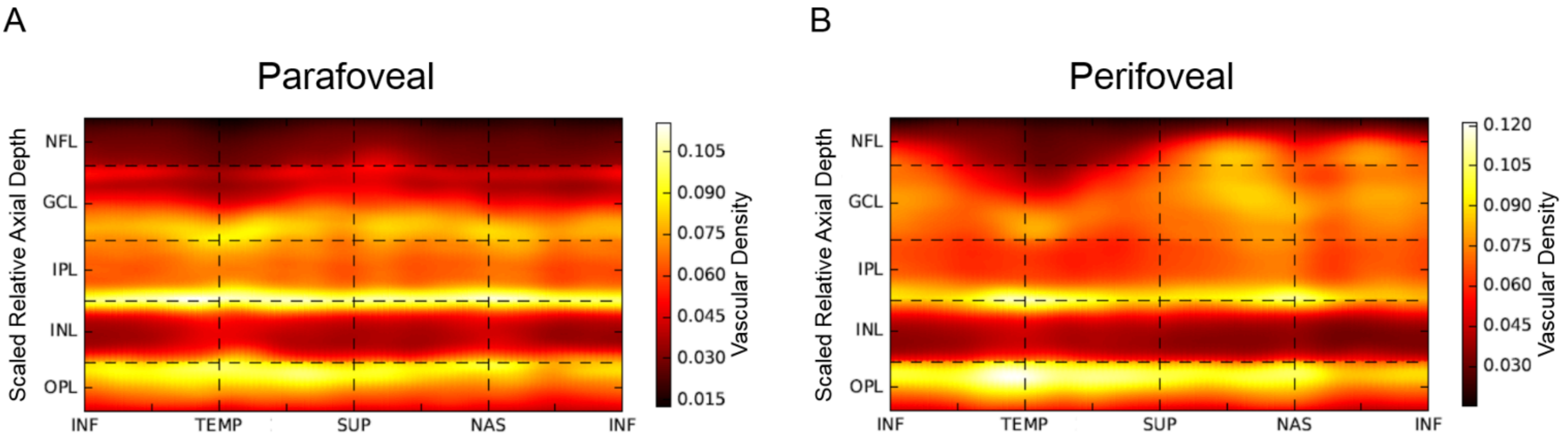

Supplemental Figure 3

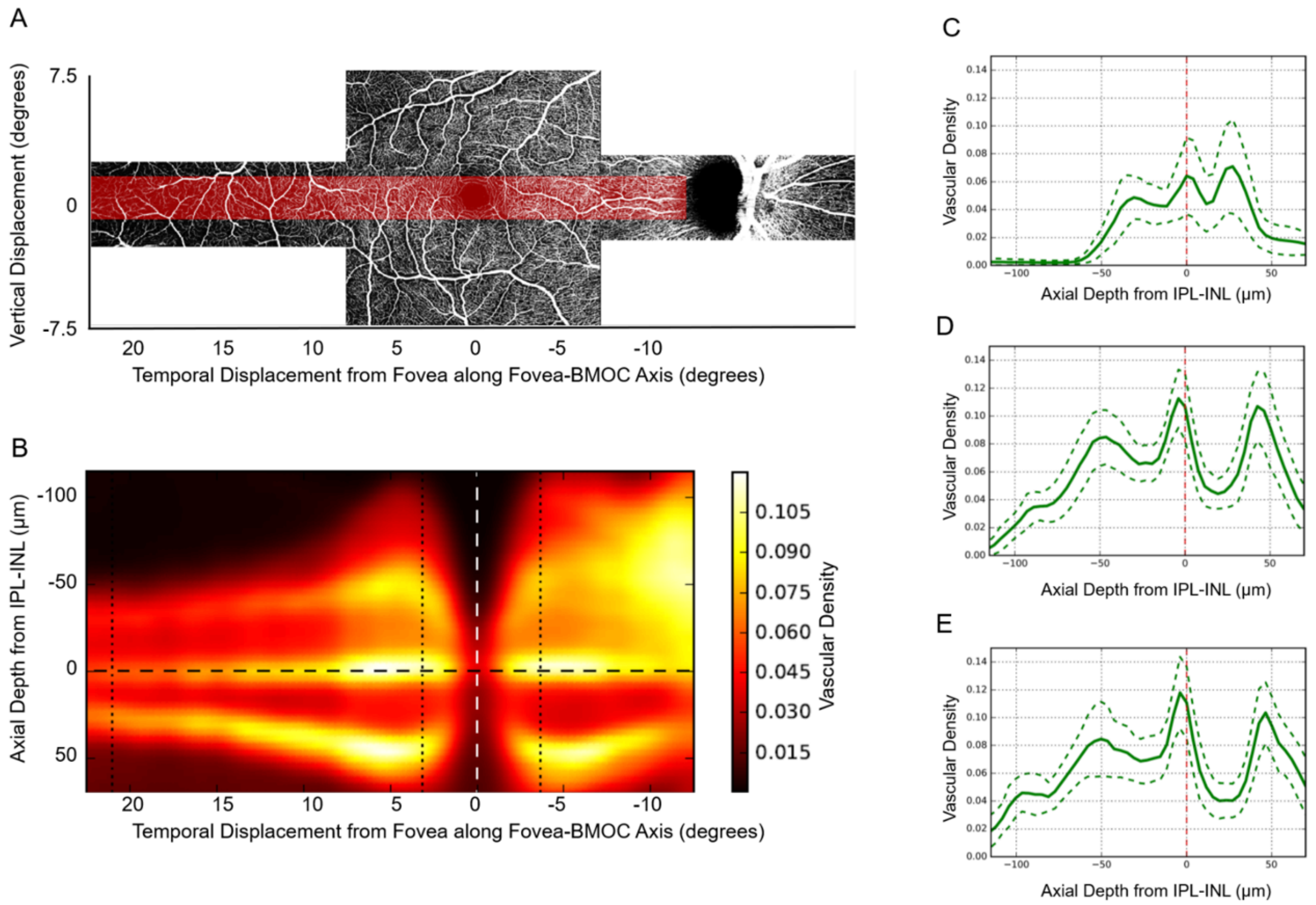

Supplement: Supplementary file 1 — Supplemental figures [file 41598_2018_27536_MOESM1_ESM.pdf]
